# Pseudoaneurysm Infection Ballooning Out of Control Following Sequential Cardiac Catheterizations: A Case Report

**DOI:** 10.7759/cureus.38721

**Published:** 2023-05-08

**Authors:** Carolina Sepulveda Ramos, Alex Tarr

**Affiliations:** 1 Internal Medicine, Nova Southeastern University Dr. Kiran C. Patel College of Osteopathic Medicine, Fort Lauderdale, USA; 2 Internal Medicine, Palmetto General Hospital, Hialeah, USA

**Keywords:** methicillin-resistant staphylococcus aureus (mrsa), pseudoaneurysm infection, methicillin resistant staphylococcus aureus bacteremia, cardiac catheterization complications, femoral artery pseudoaneurysm

## Abstract

A femoral artery pseudoaneurysm (PSA) is a potential complication of vascular access procedures, such as cardiac catheterizations, that can have serious consequences if left untreated. Although the incidence of PSA formation has decreased due to the advent of improved surgical techniques, this case demonstrates that such complications should be considered in a clinical setting. This report presents a case of right femoral PSA, pacemaker infection, and high-grade methicillin-resistant *Staphylococcus aureus* (MRSA) bacteremia status post multiple cardiac catheterizations. Treatment included open repair of his femoral artery PSA, antibiotics tailored to culture sensitivities, and pacemaker removal. The potential complications, diagnosis, management, and alternative treatment options for PSAs are discussed in order to encourage clinical awareness of a rare complication.

## Introduction

We present a case of high-grade methicillin-resistant *Staphylococcus aureus* (MRSA) bacteremia status post multiple cardiac catheterizations, propagated by right femoral pseudoaneurysm (PSA) formation and pacemaker infection. We focus on the significant risks associated with vascular access, including bacteremia, PSA, and seeding medical devices with bacteria. PSA formation is a potential risk from vascular access resulting from arterial wall disruption, especially in the setting of poor vascular wall integrity. Surgical techniques have been developed to decrease the risk of PSA formation, as they can lead to infection, thrombosis, and rupture. PSA formation may provide a pocket through which bacteria can seed and form vegetation. Additionally, implanted cardiac devices are also associated with an increased risk of bacteria seeding in the setting of bacteremia. As will be demonstrated in this case, a multidisciplinary approach should be employed when evaluating patients with extensive cardiac history.

## Case presentation

**Figure 1:**
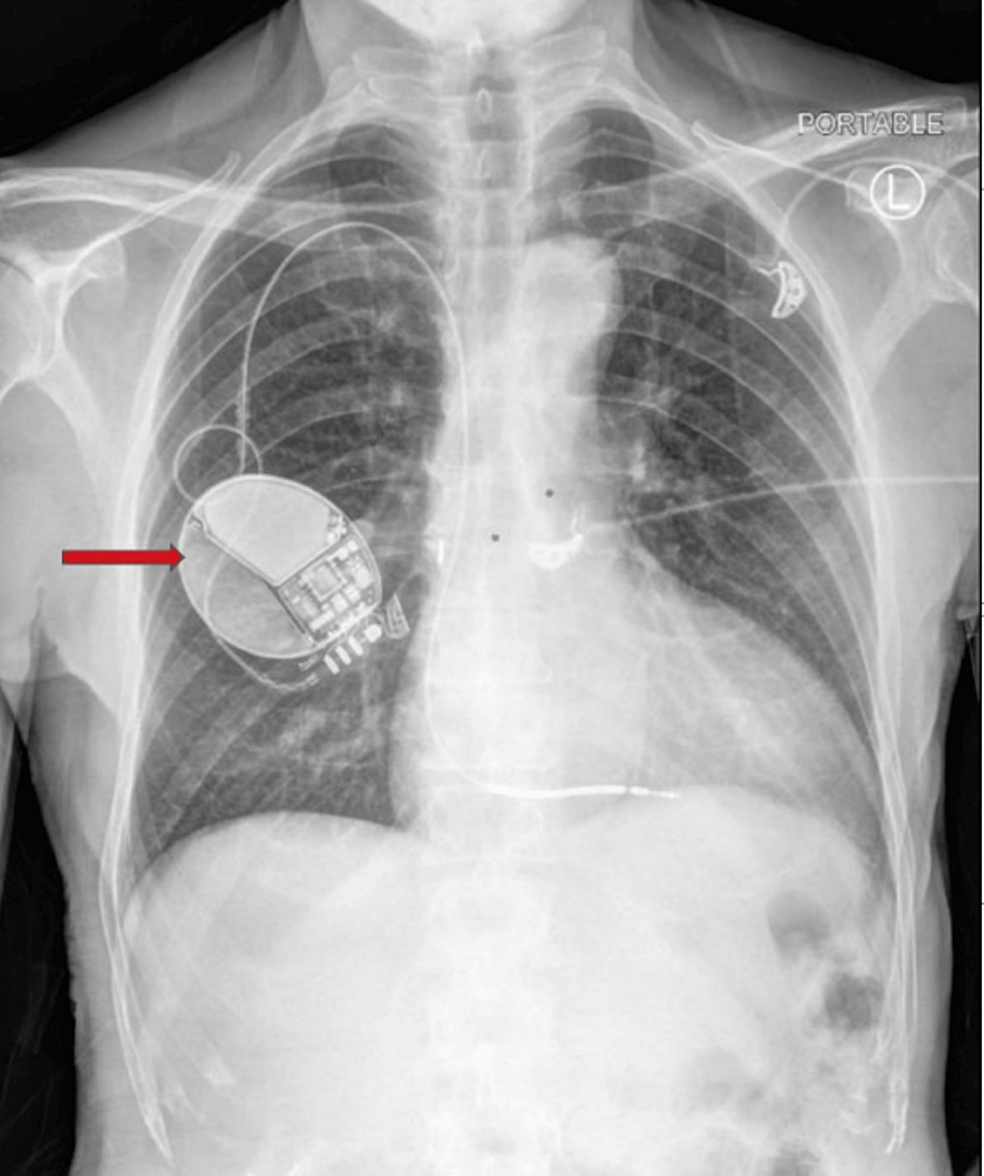
Initial chest X-ray demonstrating a right-sided automatic implantable cardioverter defibrillator (AICD).

A 59-year-old male presented to the ED status post cardiac catheterization four days prior with a complaint of chest pain radiating to his left arm. Past medical history was significant for four myocardial infarctions with two prior cardiac catheterizations without percutaneous coronary intervention (PCI), complete heart block, heart failure with a reduced ejection fraction of 15% with automatic implantable cardioverter defibrillator (AICD), and rate controlled atrial fibrillation. ECG on admission showed paced normal sinus rhythm with left bundle branch block (QRS duration <130 ms). Previous ECG was unavailable for comparison. Troponins were negative. The initial chest X-ray is as seen in Figure 1, demonstrating a right-sided AICD. Initial labs demonstrated leukocytosis, so blood cultures were collected. While the cultures were pending, the patient began experiencing fever, malaise, and fatigue. Antibiotics were started, given the patient's symptoms. When blood cultures resulted, they were positive for MRSA. Antibiotic treatment was tailored to sensitivities; however, high-grade MRSA bacteremia remained persistent. 

**Figure 2:**
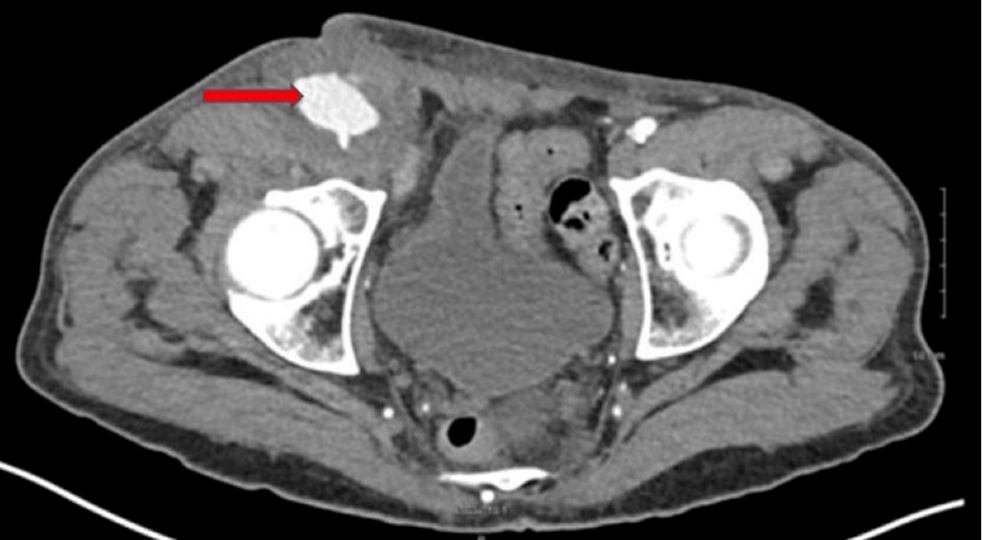
CT angiography demonstrating pseudoaneurysm formation.

A physical exam demonstrated right-sided groin swelling near his prior cardiac catheterization access site. An ultrasound of the area noted a small PSA that measured 1.4 x 1.3 cm, with no discernible neck. Further workup with CT of the abdomen demonstrated an enlarged femoral PSA with apparent right femoral artery degeneration immediately deep into the PSA (Figure 2). He underwent open repair of his femoral artery PSA. Intraoperatively, the PSA was noted to be grossly infected, and significant tissue was removed, requiring saphenous vein angioplasty and sartorius muscle flap for closure. Tissue cultures obtained during this surgery were positive for MRSA. Due to persistent MRSA bacteremia in a patient with an AICD, the patient underwent lead extraction. Cultures during this procedure were again positive for MRSA.

The transesophageal echocardiogram (TEE) did not demonstrate vegetation or signs of endocarditis. Antibiotic treatment was continued as per culture sensitivities via midline catheter. Following negative blood cultures and symptom resolution, he underwent implantation of a new AICD on the contralateral side. Following his recovery, he was discharged in stable condition with a continued antibiotic course and wound care. 

## Discussion

We present an interesting case in which a patient contracted MRSA bacteremia following multiple cardiac catheterizations propagated by infrequently seen complications related to vascular access, namely bacteremia, PSA formation, and seeding of medical devices. The point of entry for vascular access is decided depending on the procedure, the patient's presentation, past history, and anatomical concerns. For cardiac catheterization, typical access sites are either the radial or femoral arteries [1-3]. Infection is a well-known risk of access for endovascular procedures and indwelling lines [3]. The use of appropriate sterile technique, as occurs during cardiac catheterization, has made the development of bacteremia a rare complication but has not eliminated the risk [4]. PSA formation is also a possible complication due to the disruption of the arterial wall at the access site, as seen in this case. A PSA is a condition where a sac-like structure forms outside the arterial wall after an injury or puncture [5]. The pathophysiology of a PSA involves disruption of the arterial wall, which triggers an inflammatory response that activates platelets, initiates clot formation, forms a hematoma, and subsequently results in a PSA [5]. In some cases, the clot is not sufficient to contain the hematoma, leading to expansion and erosion into the arterial wall. The arterial wall responds by forming a fibrous capsule around the hematoma, which becomes the wall of the PSA [5]. The mechanism of injury, in this case, is related to direct trauma to the arterial wall during the procedure, as well as underlying arterial disease and coagulopathy. Puncture sites have varying degrees of associated risk related to PSA formation, with femoral access procuring less risk than brachial access [1,2]. Manual compression has also been found to decrease the risk of PSA formation [2,6]. Early detection and treatment of a developing PSA are important to prevent progression to more serious complications, such as PSA rupture, infection, skin necrosis, and compression of anatomical structures [7]. Duplex ultrasound is the best imaging modality for detecting PSAs because it is noninvasive and cost-effective [7,8]. CT angiography may also be used as an imaging modality, as it provides more information on the anatomy of the affected area [8]. Treatment options include conservative management, compression therapy, thrombin injection, or surgical intervention, depending on the size and location of the PSA [1,7,9]. Ultrasound-guided compression repair and thrombin injection are preferred treatment options for non-complicated PSAs; however, surgical intervention is indicated in PSAs that are infected, progressively growing, compressing underlying structures, or causing skin necrosis [7,10]. In summary, a femoral artery PSA is a potential complication of vascular access procedures that can have serious consequences if left untreated. The introduction of bacteria into the bloodstream is a well-known complication of endovascular procedures, and the patient's subsequent development of MRSA bacteremia is likely related to his prior catheterizations [11]. The development of vegetation in his PSA and on his AICD leads further illustrates the dangers of bacteremia. Seeding of implanted cardiac devices is a known risk of obtaining vascular access, and as seen in this case, may prolong hospitalization [12]. The management of patients with recurrent chest pain requires a multidisciplinary approach. In cases where coronary artery disease has been ruled out, other causes of chest pain, such as musculoskeletal pain or gastrointestinal disorders, should be considered. If catheterization is necessary, the procedure's risks and benefits should be carefully weighed, especially in patients who have already undergone multiple catheterizations [4]. Infection control measures during endovascular procedures are essential to minimizing the possibility of bacteremia development, including preoperative bathing, perioperative access site cleansing, and rigid adherence to sterile technique during the procedure [11]. Antibiotic prophylaxis may be considered in certain high-risk patients but should not be used indiscriminately [13].

## Conclusions

In conclusion, the case presented here demonstrates how repeated cardiac catheterizations may lead to PSA formation. It also highlights the importance of infection control measures during catheterization procedures, as PSAs may facilitate the seeding of bacteria despite antibiotic treatment. If there is clinical suspicion of a PSA, duplex ultrasound is an effective imaging modality that may be used to make the diagnosis. Various treatment options are available for PSAs, but treatment should be prompt to avoid PSA rupture and other complications. Additionally, the risks and benefits of repeated invasive procedures should always be considered, as in this case, repeated procedures led to medical complications and a prolonged hospital course.

## References

[1] Chen G, Wu L, Zheng L, Ding L, Wong T, Zhang S, Yao Y (2018). Combining percutaneous ultrasound-guided hematoma aspiration and compression repair to treat femoral artery pseudoaneurysm after cardiac catheterization. Int Heart J.

[2] Tamanaha Y, Sakakura K, Taniguchi Y (2019). Comparison of postcatheterization pseudoaneurysm between brachial access and femoral access. Int Heart J.

[3] Wagener JF, Rao SV (2015). A comparison of radial and femoral access for cardiac catheterization. Trends Cardiovasc Med.

[4] Ahmed I, Hajouli S (2023). Left Heart Cardiac Catheterization. https://pubmed.ncbi.nlm.nih.gov/33231993/.

[5] Tulla K, Kowalski A, Qaja E (2023). Femoral Artery Pseudoaneurysm. https://pubmed.ncbi.nlm.nih.gov/29630262/.

[6] Katzenschlager R, Ugurluoglu A, Ahmadi A (1995). Incidence of pseudoaneurysm after diagnostic and therapeutic angiography. Radiology.

[7] Webber GW, Jang J, Gustavson S, Olin JW (2007). Contemporary management of postcatheterization pseudoaneurysms. Circulation.

[8] Saleem T, D'Cruz JR, Baril DT (2023). Femoral Aneurysm Repair. https://pubmed.ncbi.nlm.nih.gov/29262227/.

[9] Shatnawi NJ, Al-Zoubi NA, Jarrah J, Khader Y, Heis M, Al-Omari MH (2019). Risk factors attributed to failure of ultrasound-guided compression for post-cardiac catheterization femoral artery pseudoaneurysms. SAGE Open Med.

[10] Schaub F, Theiss W, Busch R, Heinz M, Paschalidis M, Schomig A (1997). Management of 219 consecutive cases of postcatheterization pseudoaneurysm. J Am Coll Cardiol.

[11] Afonso E, Blot K, Blot S (2016). Prevention of hospital-acquired bloodstream infections through chlorhexidine gluconate-impregnated washcloth bathing in intensive care units: a systematic review and meta-analysis of randomised crossover trials. Euro Surveill.

[12] DeSimone DC, Sohail MR (2016). Management of bacteremia in patients living with cardiovascular implantable electronic devices. Heart Rhythm.

[13] Chehab MA, Thakor AS, Tulin-Silver S (2018). Adult and pediatric antibiotic prophylaxis during vascular and IR procedures: A society of interventional radiology practice parameter update endorsed by the Cardiovascular and Interventional Radiological Society of Europe and the Canadian Association for Interventional Radiology. J Vasc Interv Radiol.

